# Supplementary material for: Lipid polarity gradient formed by ω-hydroxy lipids in tear film prevents dry eye disease
Source: eLife. 2020 Apr 7;9:e53582. doi: 10.7554/eLife.53582 (PMC7138607; doi:10.7554/eLife.53582)
Supplement: Supplementary file 1. [file elife-53582-supp1.docx]

**Supplementary file 1. List of oligonucleotides used.**

| Primer name | Oligonucleotides |
| --- | --- |
| p1 | 5'-GGATCCTTGTGATTGGTGGATGGACAGATAG-3' |
| p2 | 5'-TCATGTCTCTGTCACTCTGACTCCTCTGC-3' |
| Involucrin-Tg-F | 5'-CTAGGAGGACCTTTCTCTGC-3' |
| Cyp4f39-F | 5'-AGCATCTACGGGACCCACCACAACC-3' |
| Cyp4f39-R | 5'-TTGCGACAGGTAATGCGGAAGTCAC-3' |
| Cyp4f39-R2 | 5'-TGAGGGTAGAGGCTCTACATTGAGC-3' |
| Awat1-F | 5'-GGAGAAACAGAGGTATATGACCAGG-3' |
| Awat1-R | 5'-TCACAAGAATATCAGCTTCTGGGTGT TGG-3' |
| Awat2-F | 5'-GGAGAGACAGACCTCTATGACCAGC-3' |
| Awat2-R | 5'-TCAAACTATCACCAGCTCCTGGGTC-3' |
| Far1-F | 5'-GATAATGTCAATATGTTAATGAACC-3' |
| Far1-R | 5'-TCAGTATCTCATAGTGCTGGATGCTCG-3' |
| Far2-F | 5'-TCCATGCTGGAGTATTTCATCAACC-3' |
| Far2-R | 5'-TTGAACAAGGGACAAATGAAGAACC-3' |
| Soat1-F | 5'-GCCGTCTTCGCCCTGTCGGCTGTGG-3' |
| Soat1-R | 5'-CTAAAACACGTACCGACAAGTCCAGG -3' |
| Hprt-F | 5'-GCTGACCTGCTGGATTACATTAAAG-3' |
| Hprt-R | 5'-CTTAACCATTTTGGGGCTGTACTGC-3' |
