# Supplementary material for: Lipid polarity gradient formed by ω-hydroxy lipids in tear film prevents dry eye disease
Source: eLife. 2020 Apr 7;9:e53582. doi: 10.7554/eLife.53582 (PMC7138607; doi:10.7554/eLife.53582)
Supplement: Supplementary file 2. [file elife-53582-supp2.docx]

**Supplementary file 2. Selected *m/z* values for AMPP-OAHFAs in MS/MS analysis.**

| ω-OH FA/FA | Precursor ion (Q1)  [M + AMPP]^+^ | Product ion (Q3)  [M + AMPP−(FA−OH)−H_2_O]^+^ |
| --- | --- | --- |
| ω-OH C28:0/C16:0 | 845.7 | 589.2 |
| ω-OH C30:0/C16:0 | 873.7 | 617.2 |
| ω-OH C32:0/C16:0 | 901.7 | 645.3 |
| ω-OH C34:0/C16:0 | 929.8 | 673.3 |
| ω-OH C36:0/C16:0 | 957.8 | 701.3 |
| ω-OH C28:1/C16:0 | 843.7 | 587.2 |
| ω-OH C30:1/C16:0 | 871.7 | 615.2 |
| ω-OH C32:1/C16:0 | 899.7 | 643.2 |
| ω-OH C34:1/C16:0 | 927.8 | 671.3 |
| ω-OH C36:1/C16:0 | 955.8 | 699.3 |
| ω-OH C28:0/C16:1 | 843.7 | 589.2 |
| ω-OH C30:0/C16:1 | 871.7 | 617.2 |
| ω-OH C32:0/C16:1 | 899.7 | 645.3 |
| ω-OH C34:0/C16:1 | 927.8 | 673.3 |
| ω-OH C36:0/C16:1 | 955.8 | 701.3 |
| ω-OH C28:1/C16:1 | 841.6 | 587.2 |
| ω-OH C30:1/C16:1 | 869.7 | 615.2 |
| ω-OH C32:1/C16:1 | 897.7 | 643.2 |
| ω-OH C34:1/C16:1 | 925.7 | 671.3 |
| ω-OH C36:1/C16:1 | 953.8 | 699.3 |
| ω-OH C28:0/C18:0 | 873.7 | 589.2 |
| ω-OH C30:0/C18:0 | 901.7 | 617.2 |
| ω-OH C32:0/C18:0 | 929.8 | 645.3 |
| ω-OH C34:0/C18:0 | 957.8 | 673.3 |
| ω-OH C36:0/C18:0 | 985.8 | 701.3 |
| ω-OH C28:1/C18:0 | 871.7 | 587.2 |
| ω-OH C30:1/C18:0 | 899.7 | 615.2 |
| ω-OH C32:1/C18:0 | 927.8 | 643.2 |
| ω-OH C34:1/C18:0 | 955.8 | 671.3 |
| ω-OH C36:1/C18:0 | 983.8 | 699.3 |
| ω-OH C28:0/C18:1 | 871.7 | 589.2 |
| ω-OH C30:0/C18:1 | 899.7 | 617.2 |
| ω-OH C32:0/C18:1 | 927.8 | 645.3 |
| ω-OH C34:0/C18:1 | 955.8 | 673.3 |
| ω-OH C36:0/C18:1 | 983.8 | 701.3 |
| ω-OH C28:1/C18:1 | 869.7 | 587.2 |
| ω-OH C30:1/C18:1 | 897.7 | 615.2 |
| ω-OH C32:1/C18:1 | 925.7 | 643.2 |
| ω-OH C34:1/C18:1 | 953.8 | 671.3 |
| ω-OH C36:1/C18:1 | 981.8 | 699.3 |
| ω-OH C28:0/C18:2 | 869.7 | 589.2 |
| ω-OH C30:0/C18:2 | 897.7 | 617.2 |
| ω-OH C32:0/C18:2 | 925.7 | 645.3 |
| ω-OH C34:0/C18:2 | 953.8 | 673.3 |
| ω-OH C36:0/C18:2 | 981.8 | 701.3 |
| ω-OH C28:1/C18:2 | 867.7 | 587.2 |
| ω-OH C30:1/C18:2 | 895.7 | 615.2 |
| ω-OH C32:1/C18:2 | 923.7 | 643.2 |
| ω-OH C34:1/C18:2 | 951.8 | 671.3 |
| ω-OH C36:1/C18:2 | 979.8 | 699.3 |
