# Supplementary material for: Lipid polarity gradient formed by ω-hydroxy lipids in tear film prevents dry eye disease
Source: eLife. 2020 Apr 7;9:e53582. doi: 10.7554/eLife.53582 (PMC7138607; doi:10.7554/eLife.53582)
Supplement: Supplementary file 3. [file elife-53582-supp3.docx]

**Supplementary file 3. Selected *m/z* values for type 2ω/α WdiEs in MS/MS analysis.**

| Total carbon chain length and degree of unsaturation | Precursor ion (Q1)  [M + H]^+^ | Product ions (Q3) | |
| --- | --- | --- | --- |
|  |  | [M + H −(C16:1 FA−OH)−H_2_O]^+^ | [M + H−(C18:1 FA−OH)−H_2_O]^+^ |
| C52:2 | 787.7 | 561.4 | 533.3 |
| C54:2 | 815.7 | 589.4 | 561.4 |
| C56:2 | 843.8 | 617.4 | 589.4 |
| C58:2 | 871.8 | 645.5 | 617.4 |
| C60:2 | 899.8 | 673.5 | 645.5 |
| C62:2 | 927.9 | 701.5 | 673.5 |
| C64:2 | 955.9 | 729.6 | 701.5 |
| C66:2 | 983.9 | 757.6 | 729.6 |
| C68:2 | 1012.0 | 785.6 | 757.6 |
| C70:2 | 1040.0 | 813.7 | 785.6 |
| C72:2 | 1068.0 | 841.7 | 813.7 |
| C52:3 | 785.7 | 559.3 | 531.3 |
| C54:3 | 813.7 | 587.4 | 559.3 |
| C56:3 | 841.7 | 615.4 | 587.4 |
| C58:3 | 869.8 | 643.4 | 615.4 |
| C60:3 | 897.8 | 671.5 | 643.4 |
| C62:3 | 925.8 | 699.5 | 671.5 |
| C64:3 | 953.9 | 727.5 | 699.5 |
| C66:3 | 981.9 | 755.6 | 727.5 |
| C68:3 | 1009.9 | 783.6 | 755.6 |
| C70:3 | 1038.0 | 811.6 | 783.6 |
| C72:3 | 1066.0 | 839.7 | 811.6 |
