# Supplementary material for: Lipid polarity gradient formed by ω-hydroxy lipids in tear film prevents dry eye disease
Source: eLife. 2020 Apr 7;9:e53582. doi: 10.7554/eLife.53582 (PMC7138607; doi:10.7554/eLife.53582)
Supplement: Supplementary file 4. [file elife-53582-supp4.docx]

**Supplementary file 4. Selected *m/z* values for type 1ω WdiEs in MS/MS analysis.**

| FA + ω-OH FA/C16:1 FAl | Precursor ion (Q1)  [M + H]^+^ | Product ion (Q3)  [M + H–C16:1 FAl]^+^ |
| --- | --- | --- |
| C32:2/C16:1 | 729.6 | 489.1 |
| C34:2/C16:1 | 757.7 | 517.2 |
| C36:2/C16:1 | 785.7 | 545.2 |
| C38:2/C16:1 | 813.7 | 573.2 |
| C40:2/C16:1 | 841.8 | 601.3 |
| C42:2/C16:1 | 869.8 | 629.3 |
| C44:2/C16:1 | 897.8 | 657.3 |
| C46:2/C16:1 | 925.9 | 685.4 |
| C48:2/C16:1 | 953.9 | 713.4 |
| C50:2/C16:1 | 981.9 | 741.4 |
| C52:2/C16:1 | 1010.0 | 769.5 |
