# Supplementary material for: Lipid polarity gradient formed by ω-hydroxy lipids in tear film prevents dry eye disease
Source: eLife. 2020 Apr 7;9:e53582. doi: 10.7554/eLife.53582 (PMC7138607; doi:10.7554/eLife.53582)
Supplement: Supplementary file 5. [file elife-53582-supp5.docx]

**Supplementary file 5. Selected *m/z* values for Chl-OAHFAs in MS/MS analysis.**

| OAHFA moiety | Precursor ion (Q1)  [M + NH_4_]^+^ | Product ion (Q3)  [Cholesterol–H_2_O]^+^ |
| --- | --- | --- |
| C42:1 | 1018.0 | 369.4 |
| C44:1 | 1046.0 | 369.4 |
| C46:1 | 1074.0 | 369.4 |
| C48:1 | 1102.1 | 369.4 |
| C50:1 | 1130.1 | 369.4 |
| C52:1 | 1158.1 | 369.4 |
| C54:1 | 1186.2 | 369.4 |
| C42:2 | 1016.0 | 369.4 |
| C44:2 | 1044.0 | 369.4 |
| C46:2 | 1072.0 | 369.4 |
| C48:2 | 1100.1 | 369.4 |
| C50:2 | 1128.1 | 369.4 |
| C52:2 | 1156.1 | 369.4 |
| C54:2 | 1184.2 | 369.4 |
