# Supplementary material for: Lipid polarity gradient formed by ω-hydroxy lipids in tear film prevents dry eye disease
Source: eLife. 2020 Apr 7;9:e53582. doi: 10.7554/eLife.53582 (PMC7138607; doi:10.7554/eLife.53582)
Supplement: Supplementary file 6. [file elife-53582-supp6.docx]

**Supplementary file 6. Selected *m/z* values for CEs in MS/MS analysis.**

| FA moiety | Precursor ion (Q1)  [M + NH_4_]^+^ | Product ion (Q3)  [Cholesterol–H_2_O]^+^ |
| --- | --- | --- |
| C16:0 | 625.6 | 369.4 |
| C18:0 | 653.6 | 369.4 |
| C20:0 | 681.7 | 369.4 |
| C22:0 | 709.7 | 369.4 |
| C24:0 | 737.7 | 369.4 |
| C26:0 | 765.8 | 369.4 |
| C28:0 | 793.8 | 369.4 |
| C30:0 | 821.8 | 369.4 |
| C32:0 | 849.9 | 369.4 |
| C34:0 | 877.9 | 369.4 |
| C36:0 | 905.9 | 369.4 |
| C16:1 | 623.6 | 369.4 |
| C18:1 | 651.6 | 369.4 |
| C20:1 | 679.7 | 369.4 |
| C22:1 | 707.7 | 369.4 |
| C24:1 | 735.7 | 369.4 |
| C26:1 | 763.8 | 369.4 |
| C28:1 | 791.8 | 369.4 |
| C30:1 | 819.8 | 369.4 |
| C32:1 | 847.9 | 369.4 |
| C34:1 | 875.9 | 369.4 |
| C36:1 | 903.9 | 369.4 |
